# Chronic stress-induced anxiety-like behavior, hippocampal oxidative, and endoplasmic reticulum stress are reversed by young plasma transfusion in aged adult rats

**DOI:** 10.22038/IJBMS.2023.72437.15754

**Published:** 2024

**Authors:** Arshad Ghaffari-Nasab, Gonja Javani, Fereshteh Farajdokht, Mohammad Reza Alipour, Gisou Mohaddes

**Affiliations:** 1 Drug Applied Research Center, Tabriz University of Medical Sciences, Tabriz, Iran; 2 Neurosciences Research Center, Tabriz University of Medical Sciences, Tabriz, Iran; 3 Stem Cell Research Center, Tabriz University of Medical Sciences, Tabriz, Iran; 4 Department of Biomedical Education, California Health Sciences University, College of Osteopathic Medicine, Clovis, CA, USA

**Keywords:** Aging, ER stress, NADH oxidase, NADPH oxidase, Stress, Young plasma

## Abstract

**Objective(s):**

Aging and stress synergistically induce behavioral dysfunctions associated with oxidative and endoplasmic reticulum (ER) stress in brain regions. Considering the rejuvenating effects of young plasma on aging brain function, in the current study, we examined the effects of young plasma administration on anxiety-like behavior, NADH oxidase, NADPH oxidase, and ER stress markers in the hippocampus of old male rats.

**Materials and Methods:**

Young (3 months old) and aged (22 months old) rats were randomly assigned into five groups: young control (Y), aged control (A), aged rats subjected to chronic stress for four weeks (A+S), aged rats subjected to chronic stress and treated with old plasma (A+S+OP), and aged rats subjected to chronic stress and treated with young plasma (A+S+YP). Systemic injection of (1 ml) young and old plasma was performed for four weeks (3 times/week).

**Results:**

Young plasma transfusion significantly improved anxiety-like behavior in aged rats and modulated oxidative stress in the hippocampus, evidenced by the increased NADH oxidase (NOX) activity and the reduced NADPH oxidase. In addition, the levels of C/EBP homologous protein (CHOP) and Glucose-Regulated Protein 78 (GRP-78), as ER stress markers, markedly reduced in the hippocampus following the administration of young plasma.

**Conclusion:**

These findings suggest that young plasma transfusion could reverse anxiety-like behavior in stress-exposed aged rats by modulating the hippocampal oxidative and ER stress markers.

## Introduction

The brain aging process promotes cellular and molecular changes in the limbic system, especially in the hippocampus (1), characterized by several pathological events such as increased oxidative stress and low-grade inflammation (2). These changes overlap with those induced in chronic stress-subjected young animals (1). Indeed, stress-related responses are not as well controlled under physiological conditions in aged animal models as in young models (1) due to reduced emotional and functional resilience to stressful events in the human and animal brain during aging (3). Thus, chronic stress may increase the risk of psychiatric disorders such as anxiety in the geriatric population (4); although the exact mechanism by which aging could enhance emotional sensitivity to chronic stress is not well described (1). 

Chronic physical and psychological stress has been found to promote the production of inflammatory cytokines in the nervous system by activating microglia cells (5). Pro-inflammatory responses mediate mood dysregulation, possibly through oxidative stress in the brain (6). The nicotinamide adenine dinucleotide (NADH)/ nicotinamide adenine dinucleotide phosphate (NADPH) oxidase system is considered an essential enzymatic source in the generation of superoxide anions (7). NADH oxidase (NOX), known as complex I, oxidizes NADH to NAD+ and transfers electrons to the respiratory chain of mitochondria (8). A significant reduction in NOX activity has been reported during aging (9), as well as chronic stress exposure in the rat brain (10), resulting in reactive oxygen species (ROS) generation and mitochondrial damage (11). On the other hand, NADPH oxidase mediates the stress-induced ROS accumulation in the aged hippocampus, and its up-regulation may lead to increased sensitivity to stress during aging. Thus, stress and aging synergistically increase NADPH oxidase levels in the hippocampus region (1). 

The endoplasmic reticulum (ER) is vital in synthesizing, folding, and transferring proteins in neuronal cells. This organelle could modulate inflammation and oxidative stress and shows an adaptive response to cellular stress (12). It has been demonstrated that chronic ER stress in the hippocampus, amygdala, and striatum may be involved in mood disorders’ pathophysiology (13). Aging-associated processes impair the folding and degradation of proteins by ER, a common feature of age-related and neurodegenerative diseases (14). Moreover, under chronic stress conditions, decreased ATP and oxidative stress levels may lead to ER dysfunction and, ultimately, improper folding of proteins (15). Chronic stress increases the expression of ER stress biomarkers such as Glucose-Regulated Protein 78 (GRP-78) and C/EBP homologous protein (CHOP) in the hippocampus, which can leak into the cytoplasm and initiate pro-inflammatory responses and oxidative stress (16).

Since aging is associated with many changes in biological blood factors, it has been suggested that rejuvenation can be induced in the brain and other tissues by transferring young blood or plasma into the aging systemic environment (17). The results of previous studies have proposed several blood factors that regulate aging and cellular senescence. However, the mechanisms underlying the effect of these factors to promote the aging phenotype need to be better defined (18). It has been documented that systemic characteristics of the young organism could improve the function of the aged tissues at the molecular and cellular levels (19). Moreover, animal studies have shown that young plasma transfusion improves age-related cognitive and behavioral deficits (20, 21). 

In this study, we systemically administered young rats’ plasma into the chronic stress-subjected aged rats and investigated anxiety-like behaviors, NOX activity, NADPH oxidase levels, and ER stress markers in the hippocampus.

## Materials and Methods

***Ethics approval***

All experimental procedures were performed in accordance with the Guide for the Care and Use of Laboratory Animals of the National Institute of Health (8th edition, 2011) and confirmed by the local ethics committee of Tabriz University of Medical Sciences (IR.TBZMED.VCR.REC.1400.363).

***Animals housing conditions***

Thirty-two aged (22 months old, weighing 450–550 g) and eight young (3 months old, weighing 200–250 g) male Wistar rats were group-housed under standard laboratory conditions with a temperature of 25 ± 2 °C and 12/12 hr of light/dark cycle. Animals were exposed to free access to food and water except during the deprivation periods applied in the stressed groups.

***Experimental groups and treatments***

**Figure 1:**
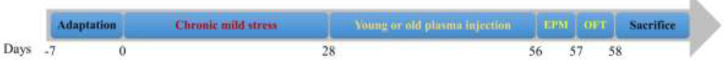
Flowchart of the experimental procedures of the study

After adaptation for one week, animals were randomly assigned into the following five groups (n = 8): 1. Young control (Y), 2. Aged control (A), 3. Aged rats receiving the chronic stress procedure (A+S), 4. Aged rats received chronic stress and were treated with old plasma (A+S+OP), and 5. Aged rats received chronic stress and were treated with young plasma (A+S+YP). The experimental design, including the procedures and time scales, is presented in Figure 1.

***Chronic stress protocol***

Chronic stress was applied as described in previous studies (22). Stressed groups were exposed for four weeks to different mild stressors, according to Table 1. To make it unpredictable, this paradigm was randomly scheduled over each week. Unstressed groups were kept in a separate room and received no manipulation.

***Anxiety-like behavior tests***

*Open field test (OFT)*

The OFT was carried out using a black wooden box (100 × 100 × 40 cm) with squares on the floor. The animal was placed in the center area and allowed to explore the field for 5 min freely. For the assessment of anxiety-like behavior, the number of facial grooming performed with the forepaws, as self-grooming, and the time spent in the center area of the field were recorded for each rat. Anxiety was indicated by increased self-grooming movements and decreased time spent in the center area (4).

*EPM*

Anxiety-like behavior was also evaluated in the animals by EPM. The apparatus is a plus shape consisting of two open arms (50 × 10 cm) and two closed arms (50 × 10 × 40 cm). The animal was individually positioned in the center of the maze and allowed to explore the apparatus for 5 min. The decreased time spent in the open arms and the low percentage of open-arm entries were interpreted as anxiety-like behavior (4).

*Young plasma injection*

Young and old animals’ plasma was collected from 20 young and 20 old rats. After anesthetizing with pentobarbital (60 mg/kg, IP), the blood samples were collected into heparinized tubes from the inferior vena cava. Blood samples were centrifuged at 1000 g and 5 min; then, plasma was isolated and stored at −80 °C until use. A+S+YP and A+S+OP rats intravenously received young and old plasma (1 ml) into the lateral tail veins three times/week for four weeks (21). Of note, Y, A, and A+S groups were treated with saline and the same heparin concentration. It has been revealed that the side effects of heparin are minimized in doses equal to 1/10 of its therapeutic dose (23). 

*Tissue sampling*

A day following the last treatment, animals were deeply anesthetized (90 mg/kg Ketamine plus 10 mg/kg Xylazine). After decapitation, the brain tissues were immediately removed, and the right hippocampus was isolated on a cold plate and kept at −80 °C for molecular assessments.

*Western blot analysis*

We determined the protein levels of CHOP and GRP78 by western blotting. The right hippocampus was homogenized on ice in lysis buffer (500 µl, Tris-HCL, pH=8, 0.003 gr EDTA, 0.08 gr NaCl, 0.025 gr sodium deoxycholate, 0.01 gr SDS, 1 tablet protease inhibitor cocktail, 10 µl Triton NP40(1%)) and was left for 20 min at 4 °C, then centrifuged (Eppendorf 5415 R) at 12,000×g for 10 min at 4 °C. Proteins were separated by SDS-PAGE and transferred onto PVDF membranes following electrophoresis. Nonspecific binding was blocked by 2 hr incubation of the membranes in 5% (w/v) nonfat dry milk in Tris-buffered saline. The membranes were then incubated for 2 hr at room temperature with primary antibodies against CHOP (1:500, Santa Cruz, sc-7351), GRP78 (1:500, Abcam, ab21685), and β-Actin (1:500, Santa Cruz, sc-47778) in the antibody buffer containing 1% (w/v) nonfat dry milk in TBS-T (0.05% (v/v) Tween-20 in Tris-buffered saline, then washed three times with TBS-T. Finally, incubation of blots with a secondary antibody (mouse anti-rabbit IgG-HRP; Santa Cruz, sc-2357) was done in the antibody buffer for 1 hr. Blots were visualized using the enhanced chemiluminescence (ECL) detection kit. The band intensities were quantified on the immunoblots using ImageJ software and normalized against the β-Actin protein (21).

*NADH oxidase activity assay*

NOX activity was determined by a fluorometric assay kit (ab273345, Abcam, USA) according to the manufacturer’s instructions. NOX activity assay kit couple’s oxidation of NADH by NOX and reduction of a colorless probe to a brightly colored product generating fluorescence at Excitation-emission (Ex/Em) = 535/587 nm. The fluorescence generated is directly proportional to the NOX activity in samples. Briefly, 10 mg of tissues were homogenized in 200 µl of lysis buffer on ice, then centrifuged at 10000 g and 4 °C for 10 min to remove cell debris and save the supernatant. Fifty microliters of NOX assay buffer was added to 30 μl of the sample supernatant. NOX activity was quantified by measuring fluorescence 535 nm for 30 min at room temperature. 

*NADPH oxidase assay*

The levels of NADPH oxidase were measured using the Abbexa ELISA Kit (abx256662, Abbexa, UK) according to the manufacturer’s instructions. For this purpose, 20 mg of tissues were extracted in 500 µl of phosphate buffer saline. After that, the tissues were homogenized and centrifuged at 5,000 g for 5 min. The supernatant was collected and used for reading at OD_450_, 25 °C. The data was calculated based on the standard curve. The standard curve was plotted as the relative OD_450_ of each standard solution (Y-axis) vs. the respective concentration of the standard solution (X-axis). Relative OD_450_ was calculated as follows:

Relative OD_450_ = (the OD_450_ of each well) – (the OD_450_ of Zero well) 

***Statistical analysis***

Data were analyzed using Graph Pad Prism software (GraphPad, La Jolla, CA, USA) and expressed in mean±standard error of the mean (SEM). The means were compared using one-way ANOVA and Tukey’s *post-hoc* test to detect the significant differences between experimental groups, *P*<0.05 was considered statistically significant.

## Results

***Young plasma alleviated stress-induced anxiety-like behavior in aged rats***

**Figure 2:**
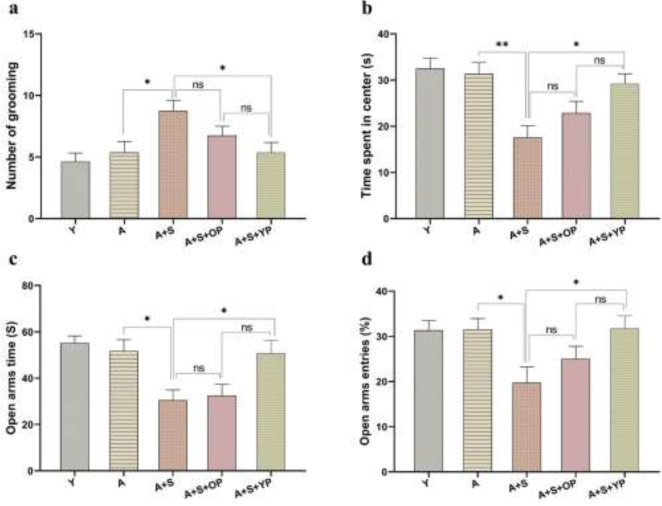
Effects of chronic stress and young plasma injection on the number of grooming episodes of aged rats

The results of OFT demonstrated significant differences in the number of grooming episodes [ F (4, 35) = 4.080, *P*=0.008] and time spent in the center [F (4, 35) = 7.043, *P*<0.001] among groups (Figure 2a, b). *Post hoc* multiple comparisons showed a significant (*P*<0.05) increase in the number of grooming episodes and a significant (*P*<0.01) decrease in time spent in the center in A+S rats compared to the aged control. However, the young plasma-treated group showed a significant (*P*<0.05) reduction in grooming and increased time spent in the center compared to the A+S group. There were no significant differences (*P*>0.05) in the number of grooming episodes and time spent in the center of the A+S+OP group compared to the A+S and A+S+YP groups.

The results of the EPM task revealed significant differences in open arms time [F (4, 35) = 6.332, *P*<0.001] and open arm entries [F (4, 35) = 3.747, *P*=0.012] among experimental groups (Figure 2c, d). *Post-hoc* analysis showed that open arms time and open arm entries were significantly (*P*<0.05) decreased in the A+S group compared to the aged control group. However, the A+S+YP group showed a significant (*P*<0.05) increase in both open arms time and the percentage of open arm entries compared to the A+S group. No significant (*P*>0.05) changes were observed in the open arm time and open arm time entries of the A+S+OP group compared to the A+S and A+S+YP groups.

***Young plasma altered hippocampal NOX activity and NADPH levels***

To elucidate whether chronic stress and young plasma affect oxidative stress, we examined NOX activity (Figure 2a) and the levels of NADPH (Figure 2b), as the two most important sources of oxidative stress, in the hippocampus of chronic stress-exposed aged rats. The results revealed significant differences in the NOX activity [F (4, 30) = 15.92, *P*<0.001] among groups. We found that NOX activity significantly (*P*<0.05) decreased in the hippocampus of the aged group compared to the young group. In addition, chronic stress exposure significantly (*P*<0.05) decreased NOX activity in the A+S group compared to the A group. However, young plasma injection significantly (*P*<0.01) increased NOX activity in the A+S+YP group compared to the A+S and A+S+OP animals. In contrast, old plasma could not alter NOX activity compared to the A+S group (*P*>0.05).

The results also revealed significant differences in the levels of NADPH [F (4, 30) = 12.64, *P*<0.001] among groups. Multiple comparisons showed that the levels of NADPH significantly (*P*<0.05) increased in the hippocampus of the aged group compared to the young group. In addition, the levels of NADPH significantly (*P*<0.01) increased in the A+S group compared to the A group. Treatment with young plasma significantly (*P*<0.05) decreased NADPH levels in the hippocampus of the A+S+YP group compared to the chronic stress-received aged animals. However, old plasma did not reverse the NADPH levels compared to the A+S group (*P*>0.05). 

***Young plasma could alter ER stress markers in the hippocampus of stress-exposed aged rats***

**Figure 3:**
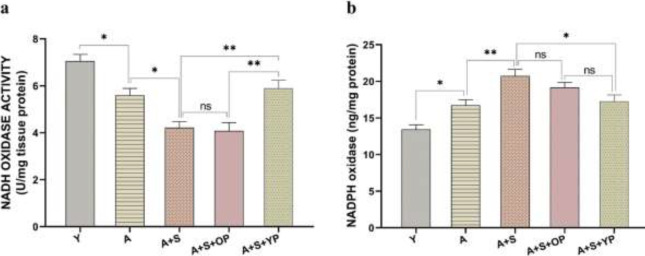
ELISA analysis of hippocampal NADPH oxidase levels and NADH oxidase activity of experimental groups

To assess the effects of young and old plasma administration on ER stress markers in the hippocampus, the expression levels of CHOP and GRP-78 were measured in the hippocampus of animal groups. As shown in Figure 3b, data revealed significant differences in the expression of CHOP protein between groups [F (4, 20) = 12.74, *P*<0.001]. *Post-hoc* analysis showed that the protein expression level of CHOP did not show significant (*P*>0.05) alteration between young and aged control groups. Chronic stress exposure significantly (*P*<0.05) enhanced the expression levels of CHOP in the A+S group compared to the A group. However, young plasma treatment significantly (*P*<0.05) reduced the protein levels of CHOP in the hippocampus of rats in the A+S+YP group compared with the A+S and A+S+OP groups. Old plasma did not significantly (*P*>0.05) alter hippocampal protein levels of CHOP compared with the A+S group.

There was also a significant interaction between groups [F (4, 20) = 10.04, *P*<0.001] in the results of the GRP-78 protein levels (Figure 3c). Tukey’s *post-hoc* test revealed that there was no significant (*P*>0.05) difference between young and aged control groups. The hippocampal GRP-78 levels were significantly higher in the A+S group (*P*<0.05) than in the aged group. This effect of chronic stress was significantly (*P*<0.05) reversed by young plasma treatment compared to the A+S group. No significant (*P*>0.05) differences were observed in the GRP-78 levels of the A+S+OP group compared to the A+S and A+S+ YP groups.

## Discussion

The present study investigated the effect of chronic transfusion of young plasma on stress induced-anxiety behavior during aging and monitored the inflammatory and ER stress markers in the hippocampus. The results revealed that repeated injection of young plasma into aged rats subjected to chronic stress markedly improved anxiety-like behavior, increased NOX activity, and reduced NADPH oxidase levels, leading to decreased ER stress markers in the hippocampus. In contrast, old plasma transfusion could not reverse chronic stress’s behavioral and biochemical effects in aged animals. Our results demonstrated that young plasma could exert an anxiolytic effect in aged animals via some possible age-related biological factors.

Stress is the most studied environmental factor that affects brain function during aging (24). On the other hand, it has been demonstrated that aging could exacerbate vulnerability to stress in the brain (25). In this regard, chronic stress has been shown to accelerate behavioral abnormalities in aged rats (26). In fact, stress and aging act in a synergistically destructive manner in the brain, especially in the hippocampus (27). Brain aging is associated with morphological, hormonal, and molecular changes resulting in loss of resilience in stressful conditions (4). Enhanced susceptibility of the aged brain in response to stress exposure could be attributed to different molecular factors (24).

Our results showed that chronic stress-induced anxiety-like behavior in the aged rats was reversed by chronic young plasma treatment. Anxiety is a serious but understudied emotional problem that could accelerate cognitive decline and other neurodegenerative disorders in the elderly (28). In addition, animal models of premature aging have demonstrated poor stress responses associated with high anxiety levels (29). Besides, an open field test has revealed anxiety-like behavior in aged male rats following chronic stress (4, 30). Inconsistent with the effects of young plasma on anxiety, previous studies have evidenced the anxiolytic effects of young plasma transfusion in aged mice (31) and cerebral amyloid angiopathy models (20).

Mitochondrial dysfunction and ROS generation may play a role in stress pathophysiology and neurodegenerative diseases (22). Lower activity of NOX is considered an index of mitochondrial functional impairment and oxidative stress development (8). The current experiment found that aging is associated with reduced NOX activity in the rat hippocampus. In addition, a reduction in NOX activity was induced by chronic stress exposure in aged rats. Evidence shows that NOX (complex I) activity is reduced with advancing age in human atria, resulting in a decline in mitochondrial respiratory capacity (9). On this basis, the NAD+/NADH ratio declines in several tissues, such as the liver and skeletal muscles and *Caenorhabditis elegans* worm during aging (32). Complex I activity is also affected by stress exposure. In this line, complex I activity is reduced in the rat brain following chronic stress exposure (10). It has been proposed that stress-induced corticosterone secretion may mediate, at least in part, the reduction of complex I activity and ROS production in the rat hippocampus (33).

Our data showed that young plasma treatment improved NOX activity in the hippocampus of aged rats subjected to chronic stress. The effects of young blood or plasma on NOX activity have yet to be studied. However, several studies have established that restoration of NAD^+^ levels could improve vascular aging phenotypes (34, 35). These data suggest that repeated injection of young plasma could improve electron transport chain function in the hippocampus by increasing NOX activity.

The current study showed that aging is associated with increased levels of NADPH oxidase in the hippocampus. Similarly, higher levels of NADPH oxidase were observed in aged rats following chronic exposure to stress, which was reversed by young plasma treatment. It has been revealed that chronic stress and the aging process commonly up-regulate NADPH oxidase levels in the hippocampus (1). In response to stress, glucocorticoids have been shown to promote oxidative stress by increasing the levels of NADPH oxidase in the hippocampus, leading to altered mood states (36, 37). Another study has documented higher levels of NADPH oxidase subunits in the hippocampus of chronic stress-subjected rats, accompanied by increased oxidative stress (38). In a pathological view, NADPH-induced ROS production is essential in oxidative damage, neuroinflammation, blood-brain barrier impairment, and neuronal death in the brain (39). Given that normal NADPH oxidase has been proposed to be crucial in physiological redox status during aging (40), the current study established that young plasma transfusion could improve age and stress-related oxidative stress via reducing NADPH oxidase levels.

It has been demonstrated that aging is associated with ER stress and the accumulation of misfolded proteins, leading to various pathological processes ranging from inflammation to apoptosis (41). Aging-associated ER stress is a common feature of neurodegenerative diseases (14). A study performed on an aging rat model has reported increased expression of NADPH oxidase and ER stress induction in the hippocampus (42). Aging-related molecular alterations in the hippocampus could interfere with the capacity to adapt to ER stress (43). ER stress is also involved in stress and mood-related pathologies as indicated by increased expression levels of CHOP protein in the hippocampus of mice model of depression (13, 16). In this regard, learned helpless rats have shown higher levels of GRP78 protein in the hippocampus region (44). Similarly, ER stress is involved in hippocampal apoptosis induced by restraint stress (45). In line with these data, the present study showed that the protein levels of CHOP and GRP-78 were up-regulated in the hippocampus of aged rats subjected to chronic unpredictable mild stress, which suggested that ER stress crucially contributed to stress and aging-induced hippocampal deficits. However, the administration of young plasma reduced the expression levels of these proteins and suppressed ER stress. 

Several lines of evidence highlighted the regenerative effects of young plasma or young systemic milieu in the hippocampus (46–48). These studies demonstrated improvement in neurogenesis, synaptic and neuronal signaling pathways, protein expression levels, and neuroinflammation in the models of aging and Alzheimer’s disease. The current study demonstrated for the first time that treatment of aged rats subjected to chronic stress with young plasma could suppress oxidative and ER stress in the hippocampus. To the best of our knowledge, the effect of young plasma on ER stress has only been investigated in the liver tissue, which has reported a significant reduction in hepatic ER stress following repeated young plasma transfusion into aged rats (49).

Since the blood-brain barrier disruption has been evident during aging, systemic alterations may affect the brain regions at cellular, structural, and functional levels (50, 51). Based on these data, it has been suggested that replacement and dilution of the old systemic milieu, which has potential pro-aging factors such as C-C motif chemokine 11 (CCL11), matrix metalloproteinase 9 (MMP9), and beta 2 microglobulin (β2M) could improve aging-related impairments in multiple tissues (52–54). On the other hand, young plasma may contain several candidate anti-aging factors, including growth differentiation factor 11 (GDF11), cadherin-13, tissue inhibitor of metalloproteinase 2 (TIMP2), thrombospondin-4 (THBS 4), SPARC-like protein 1 (SPARCL1), and oxytocin which potentially promote regenerative processes in aging tissues (54–56). Furthermore, extracellular vesicles extracted from young plasma have improved inflammaging (57) and pathological markers of Huntington’s disease (58). Further studies are needed to elucidate possible factors that might mediate young plasma’s protective and regenerative effects in aging and related disorders.

Several studies have provided evidence of young plasma transfusion’s feasibility, safety, and tolerability in human patients, suggesting its potential clinical application (31). In a human pilot study, no serious adverse effects were reported in patients with Alzheimer’s disease that received young fresh frozen plasma (59). Evidence of the anti-aging effect of dilution or exchange of aged plasma for therapeutic purposes, the FDA-approved technique, has been obtained from clinical studies (60). However, several potential risks are associated with young plasma administration in humans, such as allergic reactions, infection, and transfusion-associated circulatory overload or lung injury, especially in aged patients (48). Thus, investigating young plasma treatment’s practical advantages and effectiveness in age-related disorders requires additional human studies with a larger sample size.

**Table 1:** Duration of chronic mild stressors for a period of 1 week

Type of stressor	Duration (hr)
Intermittent white noise (85 dB)	5
Switch lights to on overnight	12
Tilt cages (45°)	7
Placing foreign objects in cages	8
Strobe lighting (300 flashes per minute)	7
Soil bedding (150 ml water in bedding)	17
Group housing	17
Food and water deprivation	22
Restricted access to food (few 45 mg pellets)	2

EPM: elevated plus maze; OFT: open field test

(a); time spent in the center zone (b); open arms time (c); and open arms entries (d) in OFT and EPM. Data are expressed as mean±SEM (n= 8). *P<0.05, **P<0.01, ns; not significant (One-way ANOVA with Tukey's post hoc analysis). Y, young control; A, aged control; A+S, aged rats subjected to chronic stress; A+S+YP, aged rats subjected to chronic stress and treated with young plasma; A+S+OP, aged rats subjected to chronic stress and treated with old plasma.

Data are presented as means±SEM (n=7/group). *P<0.05, **P<0.01, ns; not significant (One-way ANOVA with Tukey's *post hoc *analysis)

Y: Young control; A: Aged control; A+S: Aged rats subjected to chronic stress; A+S+YP: Aged rats subjected to chronic stress and treated with young plasma; A+S+OP: Aged rats subjected to chronic stress and treated with old plasma

**Figure 4:**
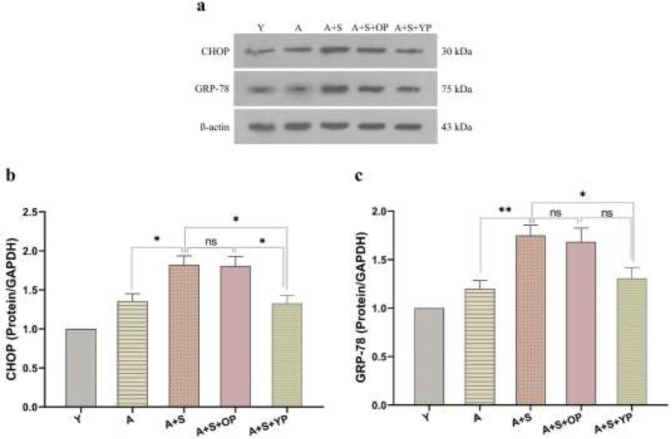
Levels of ER stress markers in the hippocampus of experimental groups of animals

A) Representative western blots; b and c) One-way ANOVA with Tukey's post hoc analysis of CHOP and GRP-78 expression. Data are presented as means±SEM (n=5/group). *P<0.05, **P<0.01, ns: Not significant. CHOP: C/EBP homolog protein; GRP: Glucose-regulated protein; Y: Young control; A: Aged control; A+S: Aged rats subjected to chronic stress; A+S+YP: Aged rats subjected to chronic stress and treated with young plasma; A+S+OP: Aged rats subjected to chronic stress and treated with old plasma

## Conclusion

The current study highlighted the roles of oxidative and ER stress in developing behavioral abnormalities in aged rats subjected to chronic stress. In addition, our data established for the first time that young plasma transfusion could reverse anxiety-like behavior as well as oxidative and ER stress markers in the hippocampus of adult rats induced by natural aging and chronic stress.

## Authors’ Contributions

A GN, G J, F F, MR A, and G M designed the experiments; A GN and G J performed experiments and collected data; F F, MR A, and G M discussed the results and strategy; G M supervised, directed, and managed the study; A GN, G J, F F, MR A, and G M approved the final version for publishing.

## Conflicts of Interest

The authors have no relevant financial or non-financial interests to disclose.
